# Exploiting machine learning for predicting skeletal-related events in cancer patients with bone metastases

**DOI:** 10.18632/oncotarget.7278

**Published:** 2016-02-09

**Authors:** Zhiyu Wang, Xiaoting Wen, Yaohong Lu, Yang Yao, Hui Zhao

**Affiliations:** ^1^ Department of Internal Oncology, Shanghai Jiao Tong University Affiliated Sixth People's Hospital, Shanghai, China

**Keywords:** machine learning, skeletal-related events, bone metastases, decision tree, support vector machine

## Abstract

The aim of the bone metastases (BM) treatment is to prevent the occurrence of skeletal-related events (SREs). In clinical, physicians could only predict the occurrence of SREs by subjective experience. Machine learning (ML) could be used as predictive models in the medical field. But there is no published research using ML to predict SREs in cancer patients with BM. The purpose of this study was to assess the associations of clinical variables with the occurrence of SREs and to subsequently develop prediction models to help identify SREs risk groups.

We analyzed 1143 cancer patients with BM. We used the statistical package of SPSS and SPSS Modeler for data analysis and the development of the prediction model. We compared the performance of logistic regression (LR), decision tree (DT) and support vector machine(SVM). The results suggested that Visual Analog Scale (VAS) scale was a key factor to SREs in LR, DT and SVM model. Modifiable factors such as Frankel classification, Mirels score, Ca, aminoterminal propeptide of type I collagen (PINP) and bone-specific alkaline phosphatase (BALP) were identified. We found that the result of applying LR, DT and SVM classification accuracy was 79.2%, 85.8% and 88.2%, with 9, 4 and 8 variables, respectively.

In conclusion, DT and SVM achieved higher accuracies with smaller number of variables than the number of variables used in LR. ML techniques can be used to build model to predict SREs in cancer patients with BM.

## INTRODUCTION

Bone is the most common site of metastasis in cancer. Cancer metastases to the bone are most prevalent among patients with advanced cancer of the breast (73%), prostate (68%), or lung (36%) [1]. Bone metastases (BM) can lead to skeletal-related events (SREs), defined as pathologic fracture, spinal cord compression, requirement for radiation, surgery to bone, and hypercalcemia [2–7]. Data from the untreated arms of clinical trials indicates that SREs are most common in patients with BM secondary to breast cancer (2-year cumulative incidence of 68%), followed by prostate cancer (2-year cumulative incidence of 49%), and non-small cell lung cancer (NSCLC) and other solid tumors (OST) (21 month cumulative incidence of 48%) [8–10]. Observational studies yielded similar patterns, with a 1-year cumulative incidence of SREs after BM diagnosis of 46% in prostate cancer patients and 38% in female breast cancer patients [11, 12].

BM and subsequent SREs can be an important burden on a cancer patients’ quality of life (QOL) and overall health status [13, 14]. SREs will dramatically reduce patients’ QOL and even shorten survival [15]. The aim of the BM treatment is to prevent the occurrence of SREs. Many risk factors play important roles in the incidence of SREs. But in clinical, physicians could only predict the occurrence of SREs by subjective experience. Multidimensional analysis of SREs requires considerable effort and expertise, demanding the development of more sophisticated ways to facilitate such complex, preferably automatic analysis [16].

Machine learning (ML) utilizes a variety of artificial intelligence and statistical models to learn from the observed data in order to create reasonable generalizations, discover patterns, classify previously unseen data or predict new directions [17]. The medical field is quickly embracing ML methodologies, such as decision tree (DT), support vector machine (SVM), as these approaches have shown progress in their usefulness in prediction and classification. Predictive models are used in a variety of medical domains for diagnostic and prognostic tasks. An increasingly large number of medical data are collected routinely, and often automatically, in many areas of medicine [18]. This implementation could prove useful in discovering ways to lower the cost of medication, improve clinical studies and help facilitate better assessments by physicians [19]. It is a opportunity for the field of ML and statistics to extract useful information and knowledge from this wealth of data [18].

ML has been used in the medical field to diagnose lung cancer, breast cancer, asthma, heart disease, dementia and other diseases and conditions. But there is no published research using supervised ML to predict SREs in cancer patients with BM. The purposes of this study were to identify the factors influencing SREs, to compare the accuracy of logistic regression(LR)-, DT- and SVM-based models in predicting SREs and to develop an effective and efficient model to predict SREs in cancer patients with BM that require intervention, based on laboratory tests commonly performed in clinical practice.

## RESULTS

### General characteristics of patients

Our study included 1143 patients with BM, median followup time was approximately 7 months. Table 2 shows the socio-demographic and clinical characteristics of the patients.

**Table 1:** Summary of attributes included in the study

Attribute	Represented as {permissible value}
Gender	Categorical { Male (1) / Female (2) }
Age	Numeric
KPS Scale	Categorical { ≥ 70 (1) / < 60 (2) }
VAS scale	Categorical { Grade 1 (1) / 2 (2) / 3 (3) }^*^
Character of BM	Categorical { Lytic (1) / Blastic (2) / Mixed (3) }
The extent of BM	Categorical { Soloway 1 (1) / 2 (2) / 3–4 (3) }**
Visceral metastases	Categorical { Without (1) / With(2) }^***^
Frankel classification	Categorical { A (1) / B (2) / C (3) / D (4) / E (5) / Without spine metastasis (6) }^****^
Mirels scale	Categorical { Without extremity metastasis (0) / 4–6 (1) / 7–9 (2) / 10–12 (3) } ^*****^
β-CTX	Numeric
N-MID	Numeric
PINP	Numeric
BALP	Numeric
CEA	Numeric
CA125	Numeric
CA153	Numeric
CA199	Numeric
AKP	Numeric
Ca	Categorical { ≥ 2.6 mmol/l (1) / < 2.6 mmol/l (2) }

* Pain level on a 10-point scale, with 0 representing no pain and 10 representing the maximum pain intensity imaginable. Grade 1: 0–3, Grade 2: 4–6, Grade 3: 7–10.

** Soloway 0 refers to patients without BM; Soloway 1 refers to patients with < 6 BM; Soloway 2 refers to patients with < 20 BM; Soloway 3 refers to patients with > 20 but less than a “super scan”; Soloway 4 refers to patients with “super scan” that is defined by a > 75% involvement of the ribs, vertebrae, and pelvic bones [19].

*** Visceral metastases defined as distant metastases, except for BM, including brain metastases.

**** Frankel classification of spinal cord injury [20]: Class A representing complete paralysis, Class B representing sensory function only below the injury level, Class C representing incomplete motor function below injury level, Class D representing fair to good motor function below injury level, and Class E representing normal function.

***** Mirels scoring system [21] based on pain intensity, site, type (lytic, mixed or blastic) and amount of bony involvement.

**Table 2:** Baseline characteristics of patients

Variables	All patients	With SREs	Without SREs
Gender (1/2)	614/529	382/240	232/289
Age (mean ± SD)	59.9 ± 23.7	58.8 ± 11.6	61.3 ± 32.8
KPS Scale (1/2)	969/174	515/107	454/67
VAS scale(1/2/3)	270/444/429	69/191/362	201/253/67
Character of BM (1/2/3)	917/83/143	517/34/71	400/49/72
The extent of BM (1/2/3)	178/610/355	108/316/198	70/294/157
Visceral metastases (1/2)	623/520	347/275	276/245
Frankel classification (1/2/3/3/5)^*^	20/20/56/58/843	20/19/47/47/418	0/1/9/11/425
Mirels (1/2/3)**	38/228/25	13/132/22	25/96/3
β-CTX (mean ± SD)	772.9 ± 488.4	810.5 ± 548.2	741.6 ± 430.2
N-MID (mean ± SD)	22.4 ± 40.1	24.4 ± 45.3	20.8 ± 35.0
PINP (mean ± SD)	166.3 ± 156.5	195.8 ± 193.3	141.7 ± 111.8
BALP (mean ± SD)	35.7 ± 25.4	35.8 ± 26.0	35.5 ± 24.6
CEA (mean ± SD)	173.4 ± 569.1	203.8 ± 499.0	148.0 ± 620.8
CA125 (mean ± SD)	171.8 ± 473.3	195.6 ± 592.6	143.3 ± 265.1
CA153 (mean ± SD)	91.1 ± 213.0	91.9 ± 205.9	90.4 ± 218.9
CA199 (mean ± SD)	299.5 ± 1034.9	347.0 ± 1196.2	259.8 ± 877.3
AKP(mean ± SD)	184.7 ± 217.0	194.4 ± 241.5	176.6 ± 194.0
Ca (1/2)	1091/52	570/52	521/0

* Among 997 patient with spine metastasis, 551 with SREs, 446 without SREs.

** Among 291 patient with extremity metastasis, 167 with SREs, 124 without SREs.

622 patients (54.4%) had SREs, 284 patients (24.8%) had developed multiple SREs, 263 patients (23.0%) had prior SREs. The rank one SREs were radiation to bone, following by pathological fracture and surgery to bone (Table 3). Most of patients suffered from lung cancer, following by breast cancer, cancer of unknown primary and prostate cancer (Table 4).

**Table 3:** Summary of SREs in the study

SREs	People	Times
Total confirmed events	973	1123
Radiation to bone	347 (35.7%)	464 (41.3%)
Pathological fracture	248 (25.4%)	263 (23.4%)
Spinal cord compression	97 (10.0%)	97 (8.6%)
Surgery to bone	229 (23.6%)	245 (21.8%)
Hypercalcemia	52 (5.3%)	54 (4.9%)

**Table 4:** Summary of cancer type and SREs in the study

Cancer type	All (%)	With SREs	Without SREs
Lung cancer	566 (49.5%)	256 (45.2%)	310 (54.8%)
Breast cancer	173 (15.1%)	115 (66.5%)	58 (33.5%)
Prostate cancer	71 (6.2%)	37 (52.1%)	34 (47.9%)
Urinary cancer	56 (4.9%)	38 (67.9%)	18 (32.1%)
Colorectal cancer	54 (4.7%)	32 (59.3%)	22 (40.7%)
Esophagus and stomach cancer	40 (3.5%)	20 (50.0%)	20 (50.0%)
Liver cancer	35 (3.1%)	29 (82.9%)	6 (17.1%)
Nasopharyngeal cancer	20 (1.7%)	12 (60.0%)	8 (40.0%)
Thyroid cancer	16 (1.4%)	11 (68.8%)	5 (31.2%)
Gynecologic cancer	16 (1.4%)	12 (75.0%)	4 (25.0%)
Cancer of unknown primary	78 (6.8%)	46 (59.0%)	32 (41.05)
Other cancer	18 (1.6%)	14 (77.8%)	4 (22.2%)

### Development of the LR model

Taking into account the 9 variables, the results of applying LR accuracy was 79.2%. VAS scale, Frankel classification, Mirels score, Gender, Cancer type, Ca, PINP, β-CTX and BALP are selected as significant variables in the LR model. A complete list of study variables in each variable set along with *p*-values are listed in Table 5.

**Table 5:** LR model of SREs in patients with BM

Variable	Coefficient	SE	*p*-value
VAS scale			
1	−3.16	0.24	< 0.01
2	−2.27	0.21	< 0.01
3	−0.43	0.18	< 0.01
Mirels score			
1	−2.58	0.89	0.24
2	−2.57	0.97	0.41
3	−2.43	0.89	0.31
Gender			
1	1.65	0.21	0.22
Cancer type			
1	−0.01	0.82	0.46
2	2.67	0.85	0.17
3	−0.04	0.87	0.96
4	0.19	0.89	0.83
5	0.48	0.88	0.58
6	0.66	0.97	0.49
7	0.93	1.00	0.35
8	−0.33	1.03	0.75
9	1.31	1.10	0.23
10	0.97	1.09	0.37
11	0.01	0.88	0.98
Frankel classification			
1	−5.86	0.40	< 0.01
2	−5.42	0.45	< 0.01
3	−4.25	0.37	< 0.01
4	−4.65	0.38	< 0.01
5	−3.29	0.52	< 0.01
PINP	−0.01	0.01	0.07
BALP	0.03	0.02	0.36
β-CTx	−0.01	0.01	0.54
Ca			
1	−1.21	0.05	0.02
Constant	5.74	0.92	0.63

SE: standard error, CI: confidence interval.

### Development of the DT model

**Figure 1:**
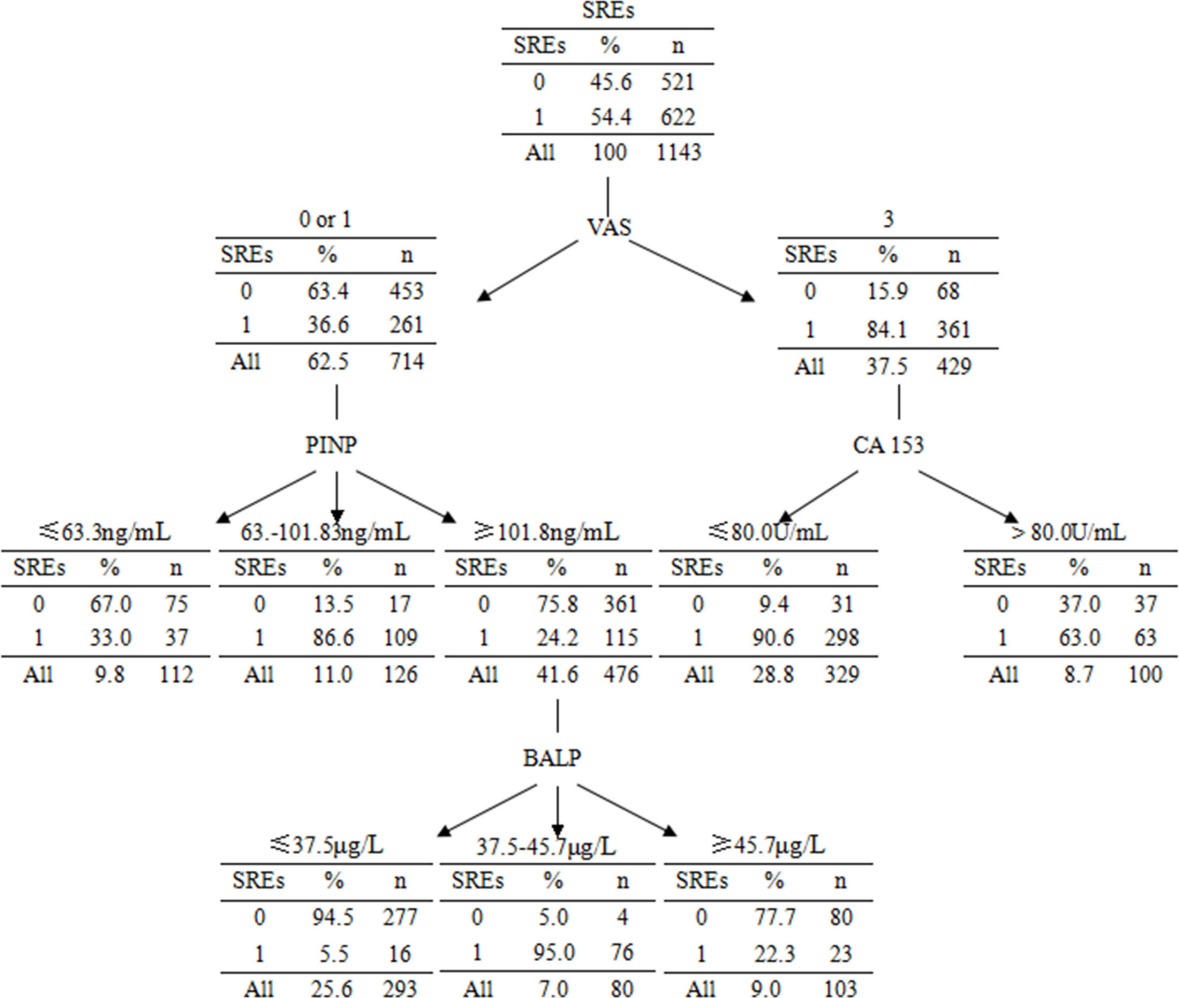
DT model of SREs in patients with BM

Figure 1 shows DT classification of SREs in patients with BM. The DT classification of SREs consisted of 4 variables which in order of importance were the following: VAS scale, PINP, CA 153 and BALP. In Figure 1, each node shows the probability of SREs for patients with BM whom are satisfied in mentioned conditions in corresponding branches.

We used CART analysis to explore high-order interactions between variables. For example, CART analysis exhibited VAS scale is the most important factor affecting SREs in patients with BM. In individuals with VAS scale Grade 3, there is an interaction with CA153, while with VAS scale Grade 1 or 2, there is an interaction with PINP, and in patients with PINP ≥ 101.8 ng/ml, there is an interaction with BALP. Figure 1 shows interactions between variables clearly.

### Development of the SVM model

To identify the variables that had the highest classification accuracy in prediction of SREs, we used SVM with radial basis function (parameter C *=* 1, γ *=* 1/number of features) that systematically searched through the space of subsets of variables, and evaluated the goodness of each variable subset according to the prediction accuracy. The variable subset showing the highest accuracy was identified as the predictor set. Parameter C is the weights between empirical error and generalization error. Parameter γ controls the shape of the separating hyperplane. It was similar to previous study on predictors of medication adherence in elderly patients with chronic disease [24].

We listed the top 8 ranked variables selected by SVM and their prediction accuracies using a combination of the top ranked variables together in Table 6 to examine the above results in detail. The accuracy using a single variable selected as VAS scale was 55.1%. The present accuracy of the SVM reached 67.4% with two variables, VAS scale and Frankel classification. The highest accuracy, 97.1%, was achieved with eight predictors: VAS scale, Frankel classification, Ca, Cancer type, Gender, Mirels score, PINP and Character of BM. The performance was very markedly decreased when more than 8 features were selected. Unlike the intuition that having more variables should achieve higher predictive performance, we found that using a small number of variables can achieve higher prediction accuracy.

**Table 6:** Combination of the top eight variables and classification accuracy in SVM

No. of variables	Combined variables in ranking order	Accuracy (%)
1	VAS scale	55.1
2	VAS scale, Frankel classification	67.4
3	VAS scale, Frankel classification, Ca	76.6
4	VAS scale, Frankel classification, Ca, Cancer type	84.3
5	VAS scale, Frankel classification, Ca, Cancer type, Gender	91.7
6	VAS scale, Frankel classification, Ca, Cancer type, Gender, Mirels score	94.4
7	VAS scale, Frankel classification, Ca, Cancer type, Gender, Mirels score, PINP	96.2
8	VAS scale, Frankel classification, Ca, Cancer type, Gender, Mirels score, PINP, Character of BM	97.1

### Comparison among prediction models

Table 7 compares the experimental results of LR, DT and SVM using 5 evaluation measures. SVM and DT showed better performance than LR in overall scoring categories, allowing identification of predictor candidates to determine the most probable SREs of a patient.

**Table 7:** Comparison between LR, DT and SVM

Model	Predicted positive	Predicted negative	Accuracy (%)	Sensitivity (%)	Specificity (%)	PPV (%)	NPV (%)
**LR**			79.2	79.4	78.9	81.8	76.3
**Positive**	494	128					
**Negative**	110	411					
**DT**			85.8	87.9	83.3	86.2	85.3
**Positive**	547	75					
**Negative**	87	434					
**SVM**			88.2	88.6	87.7	89.6	86.5
**Positive**	551	71					
**Negative**	64	457					

PPV: positive predictive value, NPV: negative predictive value.

LR showed 79.2% accuracy when all 9 variables were used for the prediction of SREs (Tables 5 and 7). Compared to the result of LR, the result of DT showed significantly higher accuracy, 85.8%, with only 4 variables on the same patient samples (Table 7). Compared to the result of LR, the result of SVM showed significantly higher accuracy, 88.2%, with 8 variables on the same patient samples (Table 7). This result indicates that ML techniques (DT and SVM) can achieve greater accuracy with a smaller number of variables than the number of variables used in LR. It is interesting to note that the most significant variable (VAS scale) selected by the DT and SVM agrees with that selected by LR.

The results of the comparison of the discriminatory power of LR, DT and SVM models are summarized in Tables 7 and 8. The AUC indicates how well a prediction model discriminates between healthy patients and patients with disease. The following guidelines have been proposed for interpretation of this area: 0.5–0.7, rather low accuracy; 0.7–0.9, moderate accuracies useful for some purposes; and > 0.9, rather high accuracy [25]. Therefore, the classification accuracies of these models were moderate.

**Table 8:** Comparison of AUC between LR, DT and SVM

Model	AUC	SE	95% CI for Exp (B)	
			Lower	Upper
LR	0.792	0.012	0.767	0.815
DT	0.856	0.011	0.835	0.876
SVM	0.882	0.010	0.861	0.900

Our results indicated that the DT and SVM model had better diagnostic capability than LR model. The AUC had achieved a moderate diagnostic power.

## DISCUSSION

Health and medical data are exponentially increasing, necessitating various means to take advantage of huge amounts of data. Big data technologies enable the fast processing of massive amounts of data [26]. Among these technologies, artificial intelligence has regained prominence as an important tool to provide intelligent services for big data, and ML techniques have also been used extensively for such purposes [27].

Traditional statistical methods, such as LR, have become increasingly difficult to use for prediction models due to several constraints that dictate the low statistical power with small sample size and complex polynomial interaction terms with curvilinear effects among the relationship of variables.

SVM and LR are similar in that both calculate a set of coefficients for variables based on a transformation of the feature space [28]. The major difference between SVM and LR is that while LR attempts to explicitly model the probability (via the odds) of outcomes, SVM attempts to directly find the best dividing hyperplane (or hyperplanes, in the case of more than two classes) regardless of the actual probability of class membership [27]. There are several advantages of SVM compared to LR. While in LR the data analyst must explicitly choose to increase the dimensionality of the feature space through the addition of interaction or polynomial terms among predictors, such transformations are standard practice in SVM approaches to classification [29]. In addition, SVM deals well with high-dimensional data, and they do not assume a parametric relationship between the model predictors and outcome.

DTs are classification algorithms which specify a “tree” of cut points that minimize some measure of diversity in the final nodes once the tree is complete. The final nodes then represent relatively homogenous individual classes [28]. To the extent that all data points classified at a given end node have a similar probability of class membership (that is, probability of treatment), then the output of DTs can be used to directly construct propensity categories [30]. Many methods for DTs (e.g. ID3, C4.5) do not provide a probability of class membership although some variants, in particular CART do provide such probabilities. However, performance of all DTs is dependent on both their method of construction and the amount of pruning (removal of highly specific nodes) performed. The major advantage of DT analysis over LR analysis is that the results of analysis are easy to understand. The simple allocation of patients into subgroups by following the flowchart form could define the predicted possibility of outcome.

In this study, ML models based on routinely available clinical and laboratory parameters were constructed for SREs prediction in cancer patients with BM. As expected, ML techniques (DT and SVM) showed greater accuracy with a smaller number of variables than the number of variables used in LR, because they establish the optimal classifier to maximize the geometric margin between samples and therefore minimize empirical classification errors. In this analysis, VAS scale was revealed as the strongest predictor of SREs. VAS scale, PINP, CA153 and BALP were selected as the predictors of SREs according to the DT model. In SVM, VAS scale, Frankel classification, Ca, Cancer type, Gender, Mirels score, PINP and Character of BM were selected as the predictors.

Most of SREs were radiation to bone, and the aim of radiation was to relieve pain. Bone surgery also had the analgesic effect. That maybe the reason why VAS scale was revealed as the strongest predictor of SREs. These findings suggested that providing appropriate analgesic therapy may reduce the occurrence of SREs. Frankel classification was designed for the assess of spinal cord compression, while Mirels score for limbs pathological fracture. These clinical factors were also revealed as the predictor of SREs. These BTMs and tumor markers were not linear with SREs. In DT, patients with moderately elevated PINP, BALP were with the highest proportion of SREs. SREs are complex phenomenons with many causes and correlates. SREs are not only related with bone formation and bone resorption, but also with the sites of BM, soft tissue mass and many other factors. Serum BTMs could only reflect bone formation and bone resorption, that maybe the reason why serum BTMs were not as well as clinical factors in the prediction of SREs.

We found that the occurrence of SREs in our study was higher than some clinical trials. The reason was because we defined percutaneous osteoplasty (PO) as bone surgery. PO can immediately restore the mechanical properties of the affected skeletal segment, provide the treated bones with increased resistance to compressive stresses, and prevent further risk of fractures, allowing immediate weight-bearing. PO can be uesed not only in vertebral metastases, but also in pelvic, iliac, and femoral metastases. PO would be effective as a combined-modality therapy for the treatment of BM [31]. We observed that suitable bone surgery, bone radiotherapy would not reduce patient's quality of life. This is just the opposite of what we defined in SREs. If there is a large clinical trial results can support this hypothesis, it will have a great impact on this model.

Our study is, to the best of our knowledge, the first attempt to use ML techniques to identify the influencing factors and to apply prediction models for the SREs of the cancer patients with BM as an alternative and complement to the traditional statistical approaches. We only used SPSS and SPSS Modeler to construct all the DT, SVM and LR model. As we all know, SPSS is widely used in the medical field for its user friendly. It would be easier for other physicians to use the models in SPSS than other software. ML models may open new possibilities to find health-related factors that otherwise would be hidden in traditional analysis methods. We used ML techniques as a supplement to the LR to develop prediction models for SREs risk groups. Our study can be used as data in healthcare for the development of new clinical assessment and interventions for the cancer patients with BM. In other words, it would be possible to develop, specifically for the cancer patients with BM, an SREs measurement tool that helps prioritize intervention for SREs risk groups. Based on the identified influencing factors, this study could also provide guidelines for healthcare staff in caring for the cancer patients with BM and could help fine-tune and improve healthcare intervention in practice.

Identification of the risk factors associated with SREs development in cancer patients with BM is essential for formulating personalized surveillance programs. Treatment of BM aims to prevent the incidence of SREs includes orthopedic management, radiation, surgery, and systemic treatments (eg, bone-targeting agents (BTAs), endocrine therapy and chemotherapy). Our Network Meta-Analysis showed denosumab, zoledronate and pamidronate were generally effective in preventing SREs in cancer patients with BM and denosumab and zoledronate were also associated with reductions in risk of pathologic fractures and radiation compared to placebo [32]. The research was not finished when these model were found, and the model should included some decision support system. Our models can predict SREs and then direct when and what treatment should be done. With low and medium level, we would give patients BTAs; and for high level, we would give patients orthopedic management, radiation even surgery. After PO, SREs especially pathologic fracture were rarely happened in the treated bone. So PO is highly recommend in the high level patients for its highly effective and safety.

The current study has several limitations, which have to be improved for prospective studies in prediction modeling.

First, it was limited to examining the impacts of individual variables. We did not examine how each variable affects others; nor did we study the nature of direct or indirect influencing factors. In future studies, we need to study how they affect predictability by identifying the meaning and detailed univariate analysis will be needed.

Second, the classification accuracies of these models were moderate. In our study, the cross validation method used the same data as the test data and the training data. If there are enough samples in the future, we will be able to get more accurate results by ensuring that the test data and the training data are separated in advance.

In this study, we sought to assess the capacity of LR, DT and SVM models to predict SREs, with the goal of developing a more predictive profile for identifying important clinical risk facts that affect SREs recurrence. We found that ML served as an effective alternative to conventional LR in identifying the key variables to show the higher classification accuracy, thereby created valuable diagnostic programs for SREs prediction.

## MATERIALS AND METHODS

### Data collection

This cross sectional retrospective study enrolled 1143 cancer patients with BM of both sexes, recruited from Department of Internal Oncology, Shanghai Sixth People's Hospital in the period between June 2007 and June 2014. This study was approved by the ethics committee of the Sixth People's Hospital, Shanghai Jiao Tong University. The principles of the Declaration of Helsinki were followed. Written consent was obtained. The diagnosis of cancer had been made by using the standard clinical criteria.

### Feature selection and reduction

A subset of 19 features including routine laboratory workup (categorical or numerical) was used for the model building process (Table 1). The dataset was created containing 2 demographic variables (age, gender), 2 general conditional variables {Karnofsky Performance Scale (KPS) and Visual Analog Scale (VAS)}, 3 metastases variables (Character of BM, extent of BM and Visceral metastases), 2 injured variables (Frankel classification of spinal cord injury and Mirels scale), 4 bone turnover markers(BTM) {bone-specific alkaline phosphatase (BALP), N-terminal midfragment of osteocalcin (N-MID), aminoterminal propeptide of type I collagen (PINP) and β-cross-linked carboxyterminal telopeptide of type I collagen (β-CTx)}, 2 biochemical variables {alkaline phosphatase (AKP) and Serum calcium} and 4 tumor markers (CEA, CA125, CA153 and CA199). These variables were selected because they were of potential clinical importance as indicated by a panel of experts. A number of data transformation techniques have been used to format and prepare the patient records to be processed by the learning algorithms (Table 1).

### Construction of the prediction models

In this study, SPSS 19^®^ and SPSS Modeler 14.1^®^ (IBM, Armonk, NY, USA) were used to construct the DT, SVM and LR models. A *p*-value ≤ 0.05 was considered to be significant for inclusion into the model. To validate each prediction model, we used a 10-fold cross validation. In 10-fold cross-validation, the data set is divided into 10 folds with equal size. Then training is carried out with 9 and testing with 1; the process is repeated until all parts have been tested.

A binary LR was performed to determine the data set under consideration, associates each record (a patient) with the probability of SREs. Stepwise selections of the independent variables were stepwise incremented and the corresponding coefficients were computed.

We constructed the DT as classification and regression trees (CART). The approach builds a binary tree by splitting the records at each node according to a function of a single input field. The evaluation function used for splitting in CART is the Gini index [23]. One of the most critical problems in tree construction is determining an appropriate size of tree. Standard methods use a “stopping rule” to determine appropriate tree sizes.

We used SVM with radial basis function (RBF) as kernels. The “SVM” function in SPSS Modeler was used to build our SVM model with the radial basis function kernel applied as its classification method.

### Comparison between prediction models

Comparisons among LR, DT and SVM discrimination for all models were performed. Sensitivity, specificity, positive predictive value (PPV), negative predictive value (NPV), and accuracy were adopted to evaluate the performance of a model. Area under curve (AUC) was calculated to test the ability of each model to distinguish patients.

### Statistical analysis

Patients were categorized into with SREs and without SREs. Qualitative variables were expressed by number, percent and compared by chi square or fishe’ s exact test. Quantitative variables were expressed by mean and standard deviation (SD) and compared by t student. Sensitivity, specificity, PPV, NPV and accuracy were calculated subsequently.
